# Microalbuminuria risks and glomerular filtration in children with sickle cell anaemia in Nigeria

**DOI:** 10.1186/s13052-019-0720-0

**Published:** 2019-11-12

**Authors:** I. E. Ocheke, S. Mohamed, E. S. Okpe, F. Bode-Thomas, M. I. McCullouch

**Affiliations:** 1 Department of Paediatrics, Jos University Teaching Hospital, Jos, PMB 2076 Nigeria; 2 Red Cross War Memorial Children’s Hospital, University of Cape Town, Cape Town, South Africa

**Keywords:** Sickle cell anaemia, Children, Microalbuminuria, Risk, Estimated glomerular filtration rate

## Abstract

**Introduction:**

Evidence of kidney damage is observed in children with sickle cell anaemia (SCA) and this continues through adulthood with progression to severe functional impairment in some. One of the earliest features of kidney damage associated with SCA is microalbuminuria. Our objective was to determine the risk factors of microalbuminuria in these children and its relationship with estimated glomerular filtration rate.

**Methods:**

This was a cross-sectional and comparative study involving three hundred and twenty three children with SCA in steady state and equal numbers of apparently healthy age and sex matched haemoglobin AA (HbAA) control, aged 6 months to 18 years. They were consecutively recruited over a 6 month period.

**Result:**

Microalbuminuria was present in 26% of the study subjects compared with 1.85% of control *P* = 0.001). Anaemia and high estimated glomerular filtration rate (eGFR) showed strong positive correlation with microalbuminuria (OR = 3.19, CI 0.953–1.116, *p* = 0.003 and OR = 1.7, CI 1.042–1.066, *p* = 0.001 respectively). Similarly, eGFR was higher in subjects with SCA than in controls and as well as in those with microalbuminuria compared with those who do not (*p* = < 0.01).

**Conclusions:**

The two most important risk factors for microalbuminuria were anaemia and high eGFR. Age category was associated more with microalbuminuria than just age as a variable. Glomerular filtration rate was higher in children with microalbuminuria than those who do not and it was also higher in children with SCA than in control.

## Introduction

Children with sickle cell anaemia (SCA) experience high rates of morbidity and mortality to the effect that the World Health Organisation (WHO) recently recognized the condition as a public health problem [1]. Its burden varies from one geographic region to another. Nigeria currently has the largest burden of sickle cell anaemia in the world with approximately 150,000 children born annually [2]. The incidence and prevalence rates of the homozygous state (HbSS) in the country are 1:300 and 2 to 3.1% respectively [3–5].

It is known that SCA affects every organ of the body and kidney abnormalities are common findings in children with the condition, some of which manifest quite early [6]. The main trigger for these abnormalities is hypoxic injury and the sequelae in the kidney is termed sickle cell nephropathy (SCN) [7].

There are several manifestations of SCN but one of the earliest features is microalbuminuria, a pre-clinical marker of kidney damage. In the adult population with SCA, microalbuminuria has been reported in up to 68% [8]. Among children however, prevalence rates of between 15.5 to 47.1% have been reported in different populations using different methods of microalbuminuria quantifications [9–14].

Generally, microalbuminuria is a significant pre-azotemic predictor of renal failure as well as of morbidity and mortality not only in SCA [15, 16]. The presence of microalbuminuria heralds development of glomerular sclerosis and other forms of glomerulopathies.

The Kidney Diseases Outcomes Quality Initiative (KDOQI), defines microalbuminuria as urinary albumin creatinine ratio of 30 to 300 mg per gram of creatinine in the first morning urine specimen [17]. It is a result of increased permeability at the glomerular filtration barrier from damage due to inflammatory cytokines, reactive oxygen species and growth factors, all related primarily to hypoxic injury [18].

There are few reports that have characterized relationship between microalbuminuria and glomerular filtration rate in individuals with SCA and most of these are from the western countries where the burden and severity of the disease is less [19, 20].

This study therefore sought to describe the risk of microalbuminuria in children with SCA and its relationship with glomerular filtration rate.

## Subjects and methods

This was a cross-sectional and comparative study carried out at the paediatric outpatient department of the Jos University Teaching Hospital. A priori calculation to determine the minimum sample size gave a figure of 322.56 for the study. Three hundred and twenty three children therefore, with SCA who were aged 6 months to 18 years were consecutively recruited over a period of 6 months from July to December 2012. All the children were on follow up in the sickle cell anaemia clinic with previously determined genotype by haemoglobin electrophoresis. They were all in steady state [21] which meant; they had not experienced any episode of painful vaso- occlusive crisis and are in a state of well-being established by a careful history and complete physical examination, had a steady haematocrit and haemoglobin values in the last 3 months prior to recruitment. Those with any co-existing disease state such as diabetes mellitus, cardiac diseases, primary kidney diseases, malaria, obesity and HIV infection that are capable of causing microalbuminuria independently were also excluded. All female children who were menstruating and any child whose dipstick urinalysis was positive for either proteinuria or haematuria were excluded. They were all on their routine hematinic and anti-malaria prophylaxis (folic acid and proguanil), respectively.

Age and sex matched controls were apparently healthy children, who were either siblings of subjects with haemoglobin genotype AA or other children on follow-up in the outpatient department with genotype AA.

Before recruitment, a prior discussion was held with the parents/guardians of eligible subjects on the nature and scope of the study. Those who consented were then asked to bring the children early in the morning on the agreed day for specimen collection and other assessment.

An interviewer administered questionnaire was used for each child and parents/guardians pair to obtain relevant information (clinical and anthropometric) and social structure of the family.

Freshly voided urine collected from each child had a preliminary urinalysis carried out on a small aliquot obtained from the main stock. The remaining urine sample was analysed for microalbuminuria using the HEMOCUE URINE ALBUMIN ANALYSER, HMC-Al 201 THREE manufactured by M-pact Corporation, Hemocue^R^, Hemocue AB, Angelholm, Sweden. The urine creatinine was analysed using a modified kinetic Jaffe reaction on an Architect Ci8200 analyzer Abbott, Abbott IL.

Serum creatinine was measured by the modified Jaffe’s kinetic reaction [22]. The Agappe diagnostic Kits^R^, Pvt. Ltd., Emakumlum Kerala, India was used for this procedure. The estimated glomerular filtration rate was calculated using the Schwartz formular [23].

Informed consent for the study was obtained from the parents/guardians of all participants. The Research and Ethical Committee of the Jos University Teaching Hospital gave approval for the study.

All data were analysed using the EPI INFO statistical software version 3.5.1 of the Centers for Disease Control and Prevention. Continuous variables were expressed as means ± standard deviations while categorical variables were expressed as percentages. The student t-test was used to compare between subjects and controls for the means of quantitative parameters. The strength of association between dependent and independent variables were determined using linear regression. A confidence interval of 95% was used and statistical significance was set at *P* value of < 0.05.

## Result

### General and clinical characteristics of the study population

Three hundred and twenty three children with SCA were in the study group and a corresponding number in the control. In the study group, there were 158 males (49%) and 165 females (51%). Majority 228(70.6%) were children 10 years and below. Table 1 shows further characteristics of the subjects and controls.

**Table 1:** General and clinical characteristics of study and control

Characteristics	Study *n* = 323 Freq(%)	Control *n* = 323 Freq(%)	t, test	*P*-Value
Gender				
Male	158 (48.9)	156 (48.3)	0.025	0.875
Female	165 (51.1)	167 (51.7)		
Age (years)				
≤ 10	228 (70.6)	207 (64.1)	3.104	0.078
> 10	95 (29.4)	116 (35.9)		
Height	118.2 ± 30.08	124.64 ± 27.20	2.849	0.005
Weight	23.16 ± 12.48	28.40 ± 14.51	4.919	< 0.01
BMI	15.51 ± 2.66	17.29 ± 4.60	5.983	< 0.01
SBP	105.93 ± 11.1	106.89 ± 6.75	1.325	0.186
DBP	69.54 ± 7.27	69.63 ± 7.27	0.808	0.420

*BMI* Body mass index

*SBP* Systolic blood pressure

*DBP* Diastolic blood pressure

### Laboratory parameters

Table 2 shows comparative assessment of some laboratory parameters such as mean urine albumin and creatinine concentrations of the study and control populations. Both urine albumin and albumin creatinine ratio were twice higher in the study compared with the control.

**Table 2:** Laboratory parameters of the study population

Parameters	Study mean ± std	Control mean ± std	t-test	*P*-value
Urine albumin (mg/L)	57.01 ± 46.29	21.82 ± 13.18	12.99	< 0.001
Urine creatinine(g/L)	3.32 ± 4.06	1.55 ± 2.56	6.66	< 0.001
Urine albumin-creatinine ratio(mg/g)	20.05 ± 18.11	5.06 ± 4.70	12.47	< 0.001
Serum creatinine (mg/dl)	0.58 ± 0.17	0.69 ± 0.20	3.463	< 0.001
Packed cell volume (%)	26.26 ± 3.98	37.55 ± 2.90	41.16	< 0.001
eGFR (ml/min/1.73m^2^)	118.38 ± 27.51	102.94 ± 5.30	9.91	< 0.001

*eGFR* Estimated Glomerular filtration rate

### Prevalence and risks of microalbuminuria in the study populations

Microalbuminuria was present in 84(26%) children among the study group while it was present in only six (1.9%) of the control, (X^2^ = 78.14, p = < 0.001). In the 84 children with microalbuminuria, the prevalence was higher in the younger children who were aged 10 years and below, 49(58.3%) than those older than 10 years 35(41.7%), *p* = 0.004. Table 3 compared some laboratory parameters of children with SCA with microalbuminuria present or not.

**Table 3:** Laboratory parameters of children with SCA with or without microalbuminuria

Laboratory parameters	Microalbuminuria		T- test	*P*-value
	Present, mean ± sd	Absent, mean ± sd		
Urine albumin (mg/L)	114.41 ± 92.37	36.81 ± 16.44	19.28	< 0.001
Urine creatinine (g/L)	2.80 ± 1.24	3.51 ± 4.65	1.38	0.170
Urine ACR(mg/g)	48.80 ± 20.30	10.0 ± 8.10	24.59	< 0.001
Serum creatinine (mg/L)	0.50 ± 0.17	0.61 ± 0.16	5.35	< 0.001
Haematocrit (%)	24.45 ± 3.72	26.92 ± 3.89	5.06	< 0.001

*ACR* Albumin creatinine ratio

The identified risk factors for microalbuminuria are shown in Table 4. Anaemia and high eGFR are the two most important risk factors. Age category and elevated systolic hypertension were the others, (*X*^2^ = 8.212, *p* = 0.004 and *X*^2^ = 20.57, *p* = 0.017 respectively). The mean haematocrit level, (Hgb)gm/dl, in SCA children with microalbuminuria was 8.15 ± 1.24, compared with 8.97 ± 1.30 in those who do not. Prevalence of anaemia was also higher in the first decade in those with microalbuminuria (59.90%) compared with (40.1%) in the second decade. In the control population, the mean hematocrit was 12.52 ± 0.97. Microalbuminuria was three times more common in children whose mean haematocrit was 8.0 g/dl or less compared with those whose mean haematocrit was 8.67 g/dl and above (OR = 3.19, CI 0.953–1.116, *p* = 0.003).

**Table 4:** Risk factors of microalbuminuria in children with sickle cell anaemia

Risk factors	Odds ratio	95% CI	STD error	*P*-value
Age	1.0314	0.9531–1.1162	0.0403	0.4425
Anaemia	3.1913	1.481–6.8739	0.3915	0.0030
DBP	0.0000	0.000 > 1.0E12	822.129	0.9735
SBP	47,009.00	0.000 > 10E12	708.6723	0.9827
BMI	0.9672	0.8817–1.0610	0.0472	0.4803
eGFR	1.663	1.0415–1.0214	0.0056	< 0.001

### Estimated glomerular filtration rate in children with microalbuminuria

The overall mean eGFR in children with SCA was 118 ± 27.51 ml/min/1.73m^2^ compared with 102.94 ± 5.30 ml/min/1.73m^2^, in the control group, *p* = < 0.001. One hundred and six (32.8%) children in the study population were classified to have high eGFR, Table 5. None of the children in the control group had high eGFR. Of the 84 SCA children with microalbuminuria, 62 (73.8%) had high eGFR compared to 44/239 (18.4%) without microalbuminuria, (*X*^2^ = 21.432, *P* = 0.01). The risk of microalbuminuria was more than one and half times in children with high eGFR compared with those who had low or normal eGFR, (OR = 1.7, CI 1.042–1.066, *p* = 0.001).

**Table 5:** Relationship between microalbuminuria and eGFR

eGFR	Microalbuminuria		X^2^	P-value
	Present n(%)	Absent n(%)		
High =106	62 (58.5%)	44 (41.5)		
Normal =193	22 (11.4%)	171 (87.6)	21.432	< 0.001
Low =24	0 (0)	24 (100.0)		
Total	84	239	323	

*eGFR* estimated Glomerular filtration rate

*High GFR* GFR > 1 standard deviation

*Normal GFR* GFR mean for age

*Low GFR* GFR < 1 standard deviation

Anaemia and eGFR were the only risk factors associated with microalbuminuria on logistic regression (*P* = 0.003 and 0.001 respectively). By linear regression, age also was an important risk factor for microalbuminuria (regression coefficient 0.105, *p* = < 0.001).

## Discussion

The overall prevalence of microalbuminuria in children with SCA in the present study was 26%. This figure is comparable with other studies that had similar sample size and used same method of microalbuminuria determination [11, 12, 14, 24]. However, for studies that had smaller sample sizes, those who used micral strips or other forms of microalbuminuria determination and whose study populations consisted of more older subjects, our finding is at variance with theirs [9, 10, 13, 20, 25, 26].

Based on age categorization, the prevalence of microalbuminuria was higher in the first decade at 58.3%, a value comparable with 57.1 to 60% reported by other workers [10–14, 25]. By linear regression, age also correlated positively and significantly with microalbuminuria, suggesting that after adjustments, older age is indeed a risk factor; (R = 0.105, *p* = 0.033). However, by logistic regression it was not, (OR = 1.0314, CI = 0.9531–1.1162, *p* = 0.4425).

The finding of a significantly higher prevalence rate of microalbuminuria in younger children may suggest earlier onset of factors that initiate kidney damage. This finding may also have arisen from subject selection where those with obvious proteinuria on dipstick urinalysis who were older, got excluded. It has been shown that there are typical glomerular structural changes observed in children with SCA at the time when microalbuminuria becomes apparent. These include increase in glomerular size, hyper filtration, thickening of the glomerular basement membrane (GBM), expansion of the mesangium and effacement of the podocyte foot processes [27–29]. These features have been described in younger children with SCA and they are risk factors for microalbuminuria. As our finding showed, mean glomerular filtration rate was higher in the population who were in the first decade compared with those in the second decade.

The other risk factors for microalbuminuria in our study were anaemia and high estimated glomerular filtration rate. Risk of microalbuminuria was three times higher in children whose mean haematocrit was 8.0 g/dl or less compared with those with mean haematocrit of 8.67 g/dl (OR = 3.19, CI 0.953–1.116, *p* = 0.003). Anaemia remained a significant risk factor for microalbuminuria after stratification for age and gender even though these factors (age and gender) on their own were not risk factors for microalbuminuria. Our study showed that among children with microalbuminuria, the prevalence of anaemia was higher in the first decade compared with the second.

The role of anaemia in the pathophysiology of microalbuminuria in SCA children has been associated with haemolysis [24, 25]. Markers of haemolysis in children with SCA such as bilirubin, lactate dehydrogenase and low haemoglobin concentration are high in those with microalbuminuria [30]. Haemolysis is an integral pathologic process in sickle cell anaemia and also an important cause of anaemia. It has been shown that early blood transfusion would suppress sickling of red cells and thus a reduction in the incidence of haemolysis and anaemia which may prevent or reduce prevalence of microalbuminuria [9].

The mean estimated glomerular filtration rate in children with SCA in our study was significantly higher than in the control population. This finding is similar to what have been previously reported in many studies [31, 32]. Similarly, a greater proportion of children with SCA and high eGFR, based on the NKF/KDOQI [17] classification, had microalbuminuria. The risk of microalbuminuria was more than one and half times higher in children with high GFR compared with those who had low or normal value as shown in our study. This may be due to glomerular changes previously described in this population [33, 34].

There are a few limitations to this study: assessment of albumin level was done on only one early morning urine specimen collected from each of the participating subjects. A second urine albumin assessment would have shown how many of these children had persistence of albuminuria which would have eliminated one possible survival bias that is a limitation for cross-sectional studies. Secondly, estimated glomerular filtration rate (eGFR) using Schwartz formula was used. This method has been identified to over-estimate glomerular filtration rate in sickle cell anaemia due to increased tubular excretion of creatinine and often decreased muscle mass. A measured GFR would have been the ideal method. Direct measurement of GFR is quite expensive and was beyond the scope of this study. The limitations notwithstanding, this study has brought out important information about microalbuminuria and GFR in children with SCA.

## Conclusions

The prevalence of microalbuminuria in our study population was high and is similar to other studies.

Age categories was a more important determinant of microalbuminuria than just age as a variable.

Glomerular filtration rate was higher in SCA children with microalbuminuria. This finding may be related to the existence of glomerular hypertrophy at this early stage of kidney damage in these children.

The two most important risk factors for microalbuminuria were anaemia and high glomerular filtration rate. Both have significant relationship with microalbuminuria on both bivariate and multivariate analysis.

## Data Availability

There were no data used in this study other than original one collected for the research.

## Abbreviations

eGFR: Estimated glomerular filtration
HbAA: Haemoglobin AA
HbSS: Haemoglobin SS
KDOQI: Kidney diseases outcome quality initiative
NKF: National kidney foundation
SCA: Sickle cell anaemia
SCN: Sickle cell nephropathy
WHO: World health organization
